# Experimental insight into the chemical corrosion mechanism of copper with an oil-in-water emulsion solution

**DOI:** 10.1039/c8ra00432c

**Published:** 2018-03-09

**Authors:** Xudong Yan, Jianlin Sun, Yanan Meng

**Affiliations:** School of Materials Science and Engineering, University of Science and Technology Beijing Beijing 100083 China sjl@ustb.edu.cn +86 10 62333768

## Abstract

Chemical corrosion mechanism of copper in an oil-in-water (O/W) emulsion is worthy of study since it would contribute to emulsion-lubrication in a metal-working process and for copper storage. The immersion experiments were carried out and the corrosion rates were measured using the weight-loss method. Surface morphology of the copper specimen was observed using a scanning electron microscope (SEM). The compositions of the corrosive residues were analyzed using an energy dispersive spectrometer (EDS) and an X-ray photoelectron spectrometer (XPS). It was found that the corrosion rate of copper in an emulsion linearly increases and the kinetics relationship could be deduced as *D*_1_ = 2.66 × 10^−3^*t*^11.68^ at room temperature (25 °C). After 1488 h of immersion time, the corrosion products on the copper surface were determined to be Cu_2_O, CuO, Cu(OH)_2_, CuCO_3_ and Cu_2_(OH)_2_CuCO_3_, which also changed the appearance of the emulsion. During adsorption, copper is more likely to coordinate with hydroxide, carboxylate or ester anions to generate copper compounds. The surfactants were consumed and the efficiency of emulsification characteristics was lost and finally, the O/W emulsion separated into two layers, which might hint the significance of introducing an inhibitor to protect the copper surfaces.

## Abstract 2

Chemical corrosion mechanism of copper in an oil-in-water (O/W) emulsion is worthy of study since it would contribute to emulsion-lubrication in a metal-working process and for copper storage.

## Graphical abstract

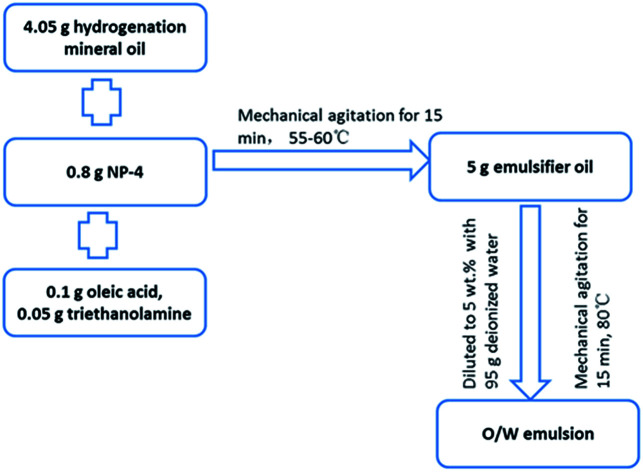

## 1. Introduction

Oil-in-water (O/W) emulsion has been seen as an attractive alternative to oil-based lubrication for hydraulic fluids,^1^ cutting fluids^2,3^ and rolling fluids^4^ since these lubricants have the advantages of cooling capabilities, reduced cleaning costs, lower toxicity and fire resistance.^5^ It can also improve the productivity through saving energy and prolonging the life cycle of the machinery.^6^ Particularly for the copper rolling process, the emulsions are used to provide lubrication at the tool-work piece interface to remove the heat generated and to improve surface finish.^7^ However, corrosion or oxidation will inevitably occur if Cu-strips are exposed to emulsions. On the one hand, complicated working environments such as stress and temperature give rise to a more corrosive tendency for copper rolling process. On the other hand, the remaining emulsion liquid on the work piece may cause rust spot, emulsion spot and other severe defects during storage.^8^ The properties of emulsions are totally different from their constituent fluids and the corrosion rates in emulsions are higher than that in individual oil and water phases.^9^ Water is a fluid that could extremely corrode a metal,^10^ while the active surfactants and emulsifier oil, which contain COO^−^ groups,^11^ –OH groups^12^ and N atoms,^13^ could make the corrosion more complex. Therefore, the corrosion behaviour of copper in emulsion is essential to be realized and the relationship of its components should be figured out.

It was observed that water occupies the major part of an O/W emulsion. Numerous studies on the corrosion behaviour of copper in water solutions have been carried out using experimental methods and various analytical tools.^14–18^ During exposure of copper to water molecules, there will be relatively strong bonds (reversible traps) between copper and hydrogen.^16^ Water could be filled with other gas compositions that affect the corrosion behaviour of the base liquid. Feng *et al.*^17^ investigated the corrosion behaviour of copper in neutral aerated simulated tap water through electrochemical methods and XPS and found that the diffusion of copper ions in oxide film controls the overall corrosion rate. The XPS spectra showed that the oxide film formed was composed primarily of cuprous oxide. Some researchers also reported that carbon dioxide (CO_2_) formed a weak carbonic acid (H_2_CO_3_) solution in aqueous media and also, it was used to lower the viscosity of oil to ease the extraction process and increase the efficiency of a well.^19,20^ In addition, copper corrosion in organic solutions such as various types of organic acids,^21–23^ fuels^24–27^ and machine oil^28,29^ has also attracted the attention of many researchers. The corrosion of copper in these organic media may be initiated by oxygen, and CO_3_^2−^, RCOO^−^ and H_2_O would further attack the Cu_2_O or CuO layer and transform it into CuCO_3_, Cu(OH)_2_·H_2_O, Cu(OH)_*x*_(RCOO)_2−*x*_, *etc.* Unfortunately, although some literature reports have considered the surface modification^30^ and barrier layer^31^ of surfactants or other emulsified components on metal surfaces, they seldom have reported the effects of corrosion behaviours of these moieties on copper surfaces, particularly for an O/W emulsion solution.

The main purpose of this study was to probe into the chemical corrosion behaviour of an O/W emulsion on a copper surface. The static immersion corrosion test was adopted for the investigation. The corrosion rate was measured using the weight-loss method and the corrosion kinetics relationship of copper was figured out at different temperatures. The corrosion mechanism of emulsions on copper surface could also be explored by analyzing the corrosion products and the erosion emulsions before and after the experiment.

## 2. Materials and methods

### 2.1 Oil-in-water emulsion preparation

**Fig. 1:**
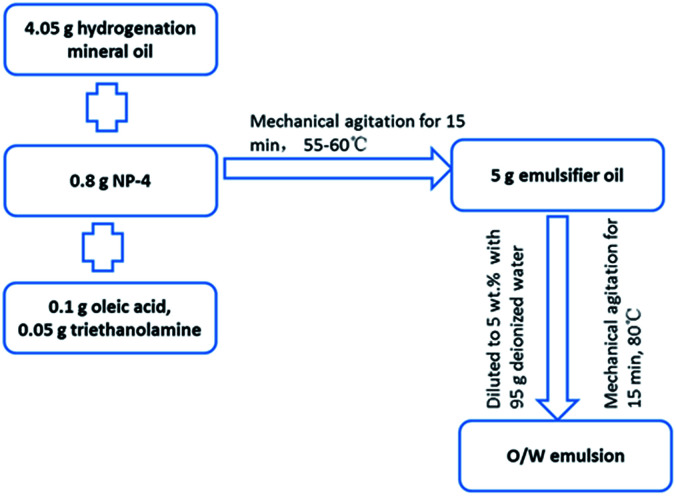
The preparation process of O/W emulsion.

The O/W emulsion in this study was composed of 5 g emulsified oil and 95 g deionized water. The emulsified oil contained base oil (81 wt% hydrogenation mineral oil, the physicochemical properties of which is shown in Table 1), an emulsifier (16 wt% NP-4 (C_9_H_19_C_6_H_4_O(CH_2_CH_2_O)_4_H)), and non-phosphorus surfactants (2 wt% oleic acid (C_8_H_17_CH=CH(CH_2_)_7_COOH) and 1 wt% triethanolamine (N(CH_2_CH_2_OH)_3_)). The preparation process of O/W emulsion is shown in Fig. 1.

**Table 1:** Physicochemical properties of hydrogenation mineral oil

Main characteristic	Parameter
Kinematic viscosity (40 °C), mm^2^ s^−1^	5.0
Flash point (open), °C	150.0
Boiling range, °C	240.0–280.0
Sulfur content (wt%)	0
Aromatic content	0.2

### 2.2 Corrosion measurements

The emulsion was treated as the corrosive solution. Circular strips (*φ* 38 mm × 1.50 mm) of pure copper (99.9 wt% in ASTM) with a *φ* 1 mm hole at the border were utilized as specimens. All the copper samples were made as to have the same initial mass and surface roughness. They were abraded and polished to obtain a mirror-like appearance, washed with distilled water, rinsed with acetone, and finally dried with N_2_. The static immersion corrosion test was carried out by immersing the copper samples in the O/W emulsion for different times. The weight of each sample was recorded prior to and after the immersion test using a balance with a four decimal accuracy. The experimental results were converted from mass loss to corrosion rate variations and were calculated using eqn (1):1
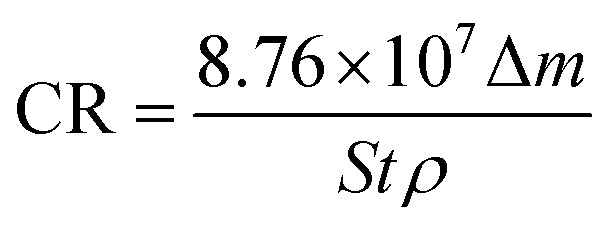
where CR is the corrosion rate (μm per year); Δ*m* represents the mass loss before and after the experiments (g); *S* stands for the superficial area of Cu immersed in an emulsion (cm^2^); *t* is the corrosion time (h); *ρ* represents the density of copper (g cm^−3^).

### 2.3 Electrochemical measurements

Strips (10.0 mm × 10.0 mm × 1.50 mm) of pure copper (99.9 wt%) were utilized as specimen electrodes for electrochemical measurements. All copper electrodes were ground up to no. 1500 and then polished to a mirror-like surface, followed by ultrasonic cleaning for 10 min with deionized water and acetone, in sequence. A typical three-electrode system consisting of a Cu working electrode, a Ag/AgCl (SCE) reference electrode and a Pt auxiliary electrode was employed to investigate the corrosion behaviour at room temperature (25 ± 0.1 °C). Potentiodynamic measurements were obtained and recorded from a potential of −2.0 V_SCE_ to 1.5 V_SCE_ at a scan rate of 1 mV s^−1^ by a VersaSTAT (MC) multichannel potentiostat system.

### 2.4 Specimen analysis and surface characterization

Surface morphology of the copper specimen was observed with a scanning electron microscope (SEM, EVO 18). The compositions of corrosive residues were analyzed using an energy dispersive spectrometer (EDS) and an X-ray photoelectron spectrometer (XPS, ESCALAB 250 Xi). The erosion emulsion was collected again and analyzed using a transmission electron microscope (TEM, G2 F20) and through Fourier transform infrared spectroscopy (FT-IR, SPECTRUM ONE), respectively. Particle sizes of the emulsion droplets were measured by a laser particle instrument (LS-POP).

## 3. Results and discussion

### 3.1 Corrosion kinetics investigation

**Fig. 2:**
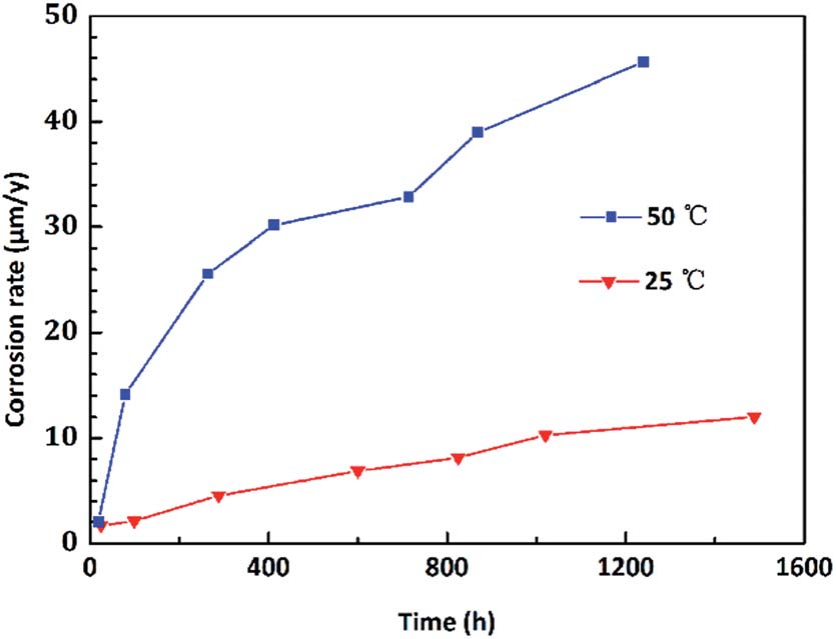
Corrosion rate of copper in an O/W emulsion for different immersion times (0–1488 h).

Fig. 2 shows the corrosion rate of copper upon exposure to an O/W emulsion from 0 to 1488 h of immersion time. The corrosion rate exhibited a nearly linear increasing trend at 25 °C and the entire corrosion process was at a low corrosion level. However, the corrosion behaviour of copper was extremely sensitive to the temperature. At 50 °C, the corrosion rate sharply enhanced as compared to that at room temperature in the first 600 h. Then, it exhibited a “steady plateau” between 600 h and 800 h. Corrosion rate varied slowly at this period, which could be attributed to the formation of the passive layer that blocked the corrosion process. After 800 h of immersion time, the corrosion rate increased again. Particularly, the corrosion rate of copper tended to zero within 24 h at both temperatures, indicating that the corrosion behaviour of copper exhibits an incubation period, which is different from other metals.

**Fig. 3:**
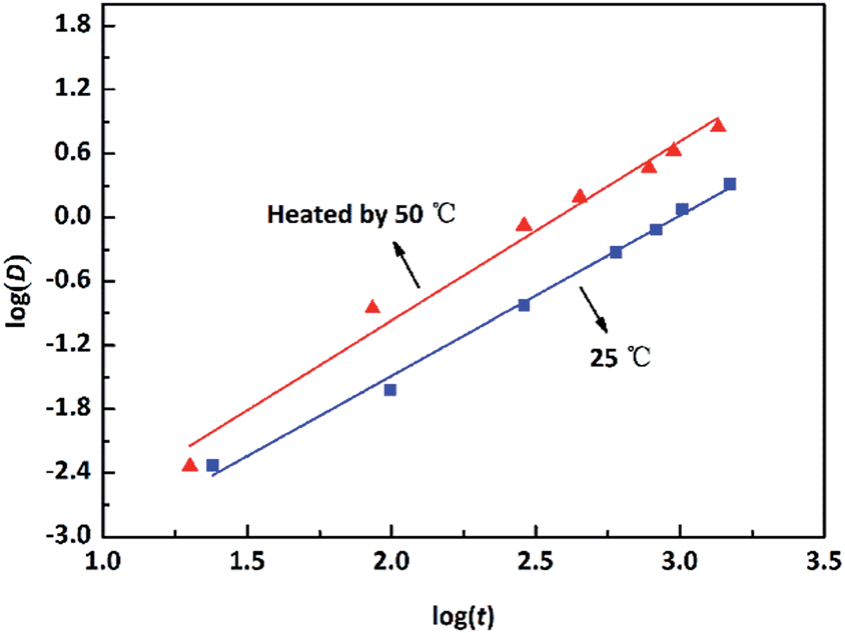
Evolution of the corrosion depths of copper in an O/W emulsion at room temperature (25 °C) and an increased temperature (50 °C).

To intuitively illustrate the corrosion degree of copper in corrosive solutions, the corrosion rate was converted to the corrosion depths. The evolution of corrosion depth in an O/W emulsion obeys a power exponent increasing relationship, which could be determined as follows:

2 *D* = *At*^*m*^

where *D* is designated as the corrosion depths (μm); *A* is a proportional constant; *t* represents immersion time (h). The relationship can be fitted in a linear curve of log(*D*) and log(*t*). The goodness of fit of this power exponent corrosion kinetics model was up to 0.98. As shown in Fig. 3, at 25 °C, it showed log(*D*_1_) = 1.6845 log(*t*_1_) − 4.3378 with a correlation index *R*_2_ = 0.984; and at 50 °C, it showed log(*D*_2_) = 1.5071 log(*t*_2_) − 4.5029 with *R*_2_ = 0.995. Therefore, the corrosion kinetics relationship of copper in emulsion for different temperatures could be deduced as *D*_1_ = 2.66 × 10^−3^*t*^11.68^ (for room temperature, 25 °C) and *D*_2_ = 1.03 × 10^−3^*t*^21.51^ (for a higher temperature 50 °C). This model could be used to quantitatively predict or evaluate the corrosion depths of copper in an O/W emulsion system.

### 3.2 Electrochemical results

**Fig. 4:**
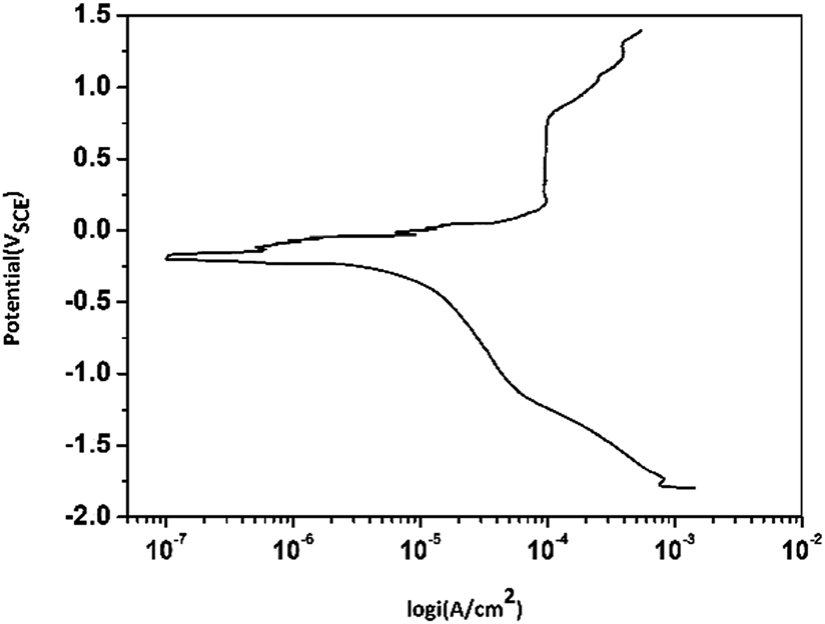
Potentiodynamic polarization curves for Cu electrodes in O/W emulsion (55 min immersion, 1 mV s^−1^).

Fig. 4 shows the polarization curves of copper immersed in the O/W emulsion. It was found that a clear passivation region appeared at potentials 0.2–0.8 V_SCE_, which indicated that the surfactants or emulsifiers were adsorbed on to copper electrodes to form a passive layer, which protects both anodic area and cathodic area from being corroded.^32,33^ However, after 0.8 V_SCE_, the passive layer was broken and potential growth with the increase in current density occurred. The corrosion current density (*I*_corr_) was around 1.0 × 10^−4^ A cm^−2^ and the corrosion potential was −189.7 V, which reflects a different corrosion tendency of Cu in the O/W emulsion as compared to that in a 3.5 wt% NaCl solution as claimed by Liu^34,35^*et al.*

### 3.3 Surface morphology and emulsion observations

**Fig. 5:**
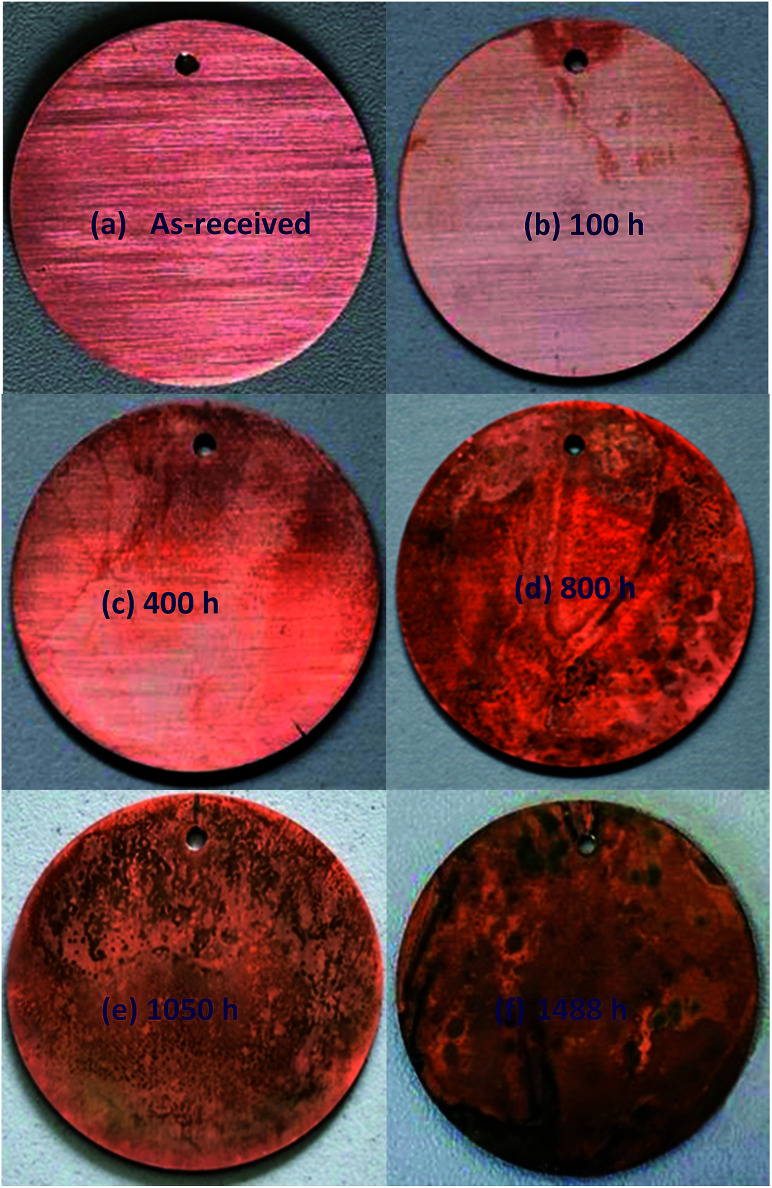
Photographs of copper surfaces upon immersing in an O/W emulsion for different corrosion times at room temperature (25 °C).

Fig. 5 shows the macroscopic morphologies of test copper specimen before (Fig. 5(a)) and after (Fig. 5(b)–(f)) immersing in an emulsion for different times. It is seen that the plowing wear on the original copper surface gradually disappears within 100 h and a brick-red layer appears at the edge of the copper surface. This layer became thick with the increase in the immersion time. When the immersion time was over 400 h, the copper surface was covered with large amounts of these brick-red corrosion products. It was further observed that the color of the corrosion product layer changed from reddish to blackish at 800 h and the corrosion layer became dense. Most of the black corrosion products peeled off after 1050 h, accompanied by the appearance of a green layer on the copper surface. The green layer ultimately covered the entire surface when the copper was immersed for 1488 h. These results demonstrate the conversion of copper compounds on the immersed surface with the increase in immersion time.

**Fig. 6:**
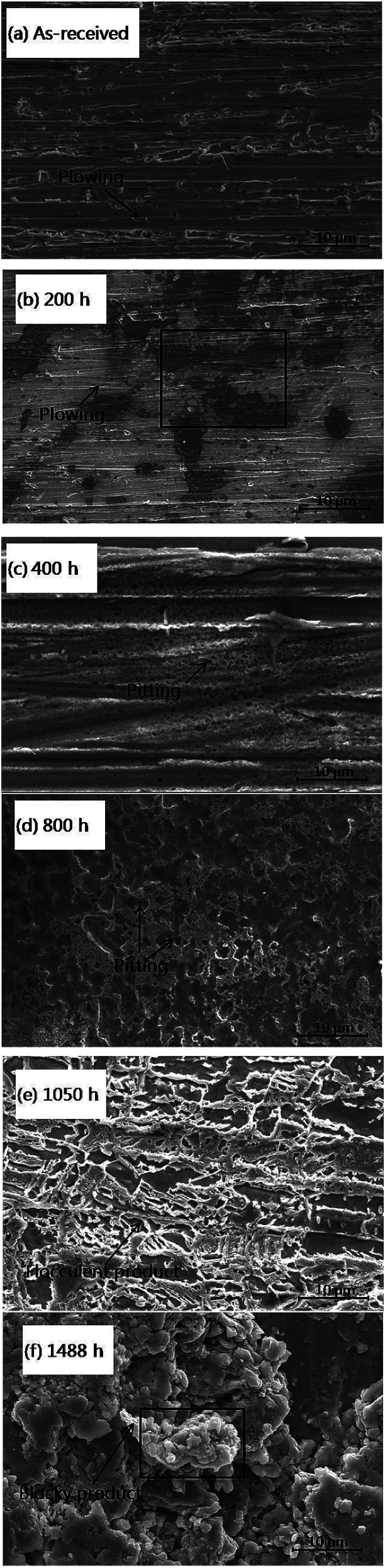
SEM images of copper surfaces that were immersed in an emulsion for different immersion times.

The immersed copper was then cleaned and SEM micrographs were recorded for further studies. As shown in Fig. 6(a) and (b), at the initial stage of corrosion, within 200 h of immersion time, the polished plowing trace gradually disappeared and the entire surface tended to become smooth. A number of small pits are observed to be formed randomly on the copper surface after 400 h of immersion time (Fig. 6(c)). The average size of these pits is less than 1 μm. The size of the corrosive pits increased and became dense when the immersion time reached 800 h (Fig. 6(d)). The pits grew to 2–3 μm in average size and appeared as alveolate corrosion products on the copper surface. After 1050 h of immersion time (Fig. 6(e)), a mass of new flocculent products was generated and the surface morphologies changed loosely and became contiguous. Finally, after over 1488 h (Fig. 6(f)) of immersion, former corrosion products aggregated and evolved in the form of massive blocky products in the electron microscopic morphologies. The size of these corrosion products were larger than 20 μm in average.

**Fig. 7:**
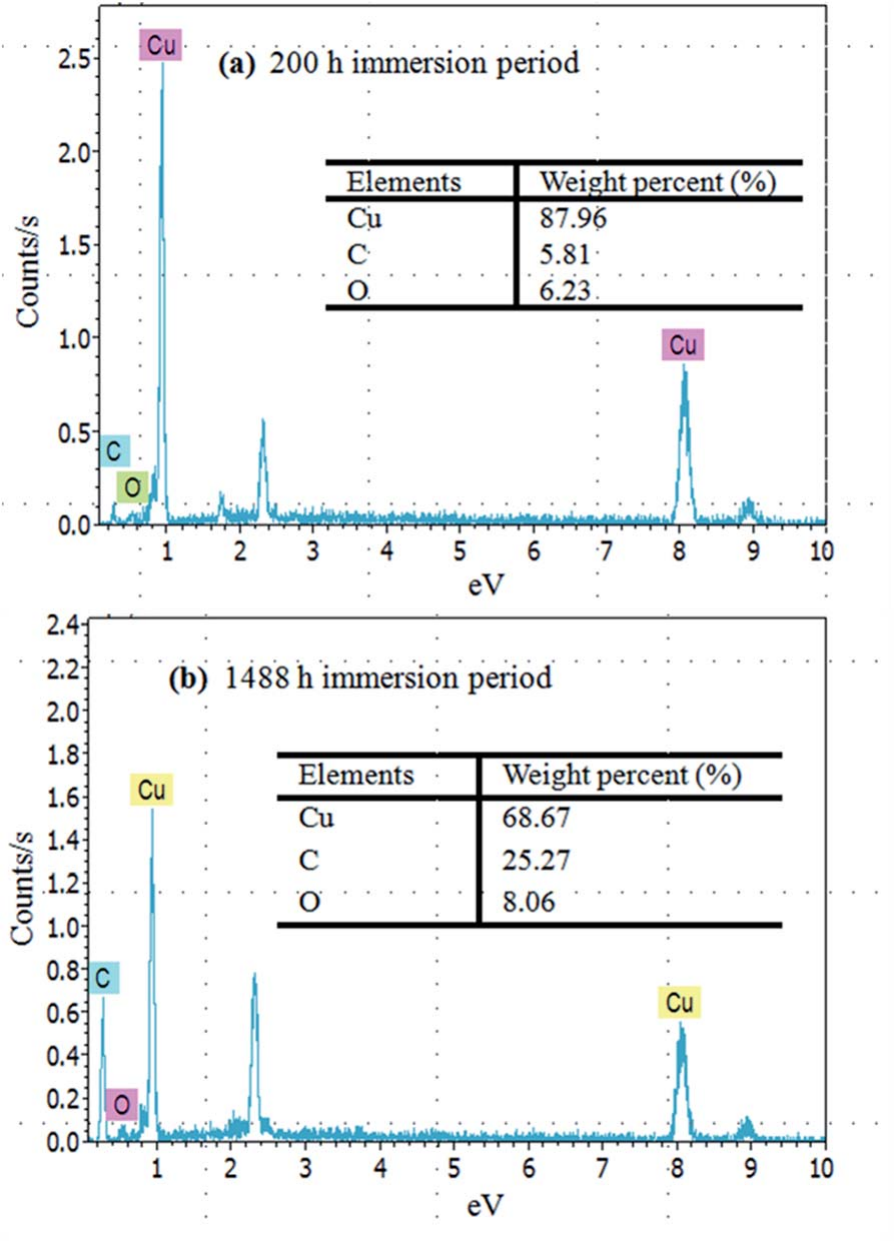
EDS analysis of copper surfaces for (a) 200 h and (b) 1488 h immersion times.

Elemental analysis of the immersed copper surface indicated the presence of carbon and oxygen. In the case of the initial corrosion period, within 200 h (Fig. 7(a)) of immersion, the EDS showed only 5.81% C, 6.23% O and a large amount of Cu (87.96%) on the copper surface. However, with the increase in the immersion time, the oxygen and carbon contents improved. In particular, after over 1488 h (Fig. 7(b)) of immersion time, the content of C and O rose to 25.27% and 8.06%, respectively, implying that the corrosion products were present as copper oxides or copper carbon oxides. Even after cleaning, the chemisorptions between copper surface and emulsion could generate stable corrosion products.

**Fig. 8:**
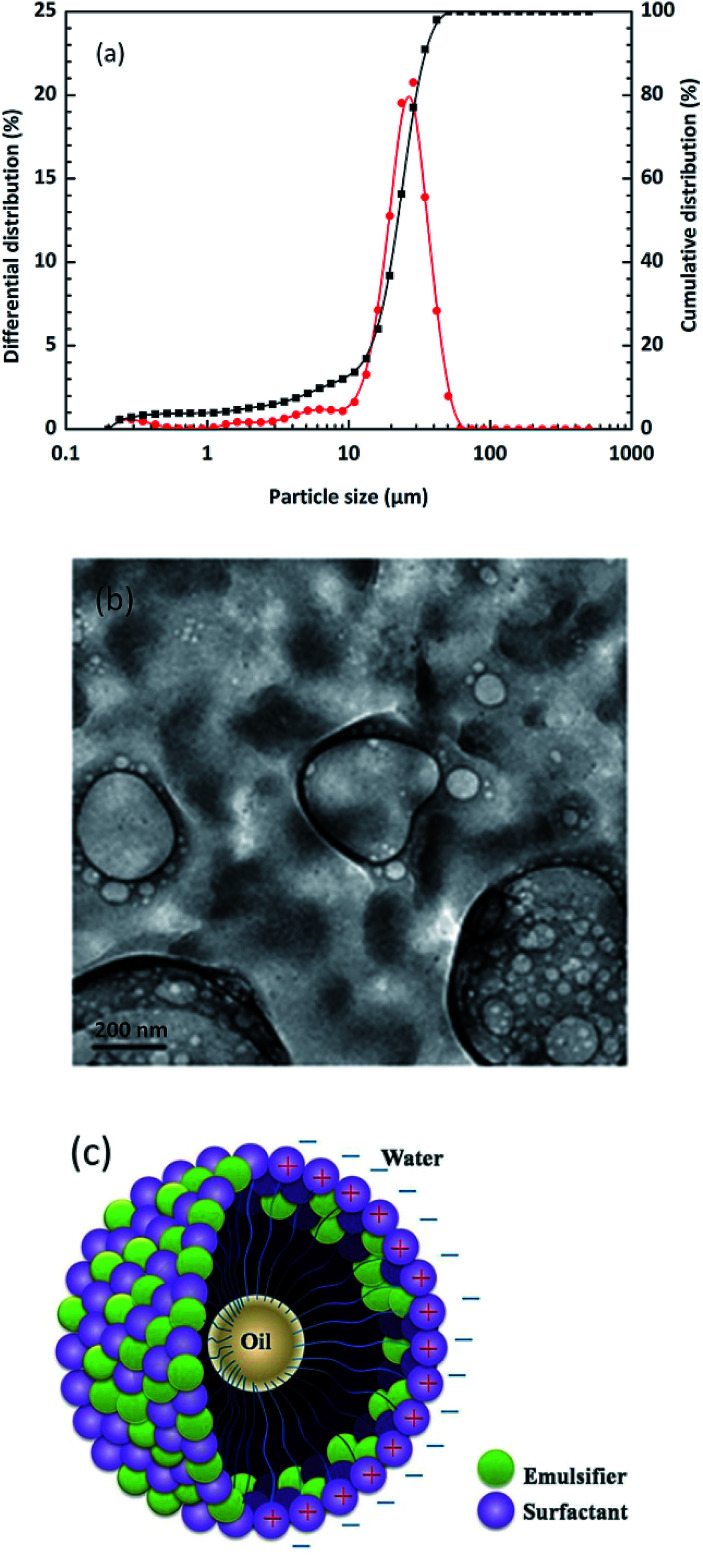
(a) Differential and cumulative distribution of particle sizes of emulsion droplets, (b) TEM images of emulsion droplets for 800 h immersion time at 25 °C and (c) the formation schematic diagram of an O/W emulsion.

It is possible that the addition of oil-phase solvents to the aqueous phase, which can change the charging behaviour of particles, might also improve the capacity of these particles to stabilize emulsions.^36^ Many factors influence the particle size of emulsion droplets such as concentrations, the type of surfactants, the preparation process and the employed environment. Also, the stability of an emulsion is strictly controlled by the particle size of the emulsion droplets. As shown in Fig. 8(a), particle sizes vary from 10 to 50 μm with an average of 22 μm, which nearly obeys normal distribution. It can be also seen from TEM morphologies (Fig. 8(b)) that the emulsion droplets appeared spherical and elliptical. These observations corresponded to those in the formal SEM images. With the help of surfactants and emulsifiers, the small-sized water molecules seemed to encapsulate the large-sized oil phase in order to form a stable system (Fig. 8(c)). These results further illustrate that the pits were the main adsorption area of emulsion droplets, which were also important in the formation mechanism of “emulsion spot” defects in the actual copper rolling process.

**Fig. 9:**
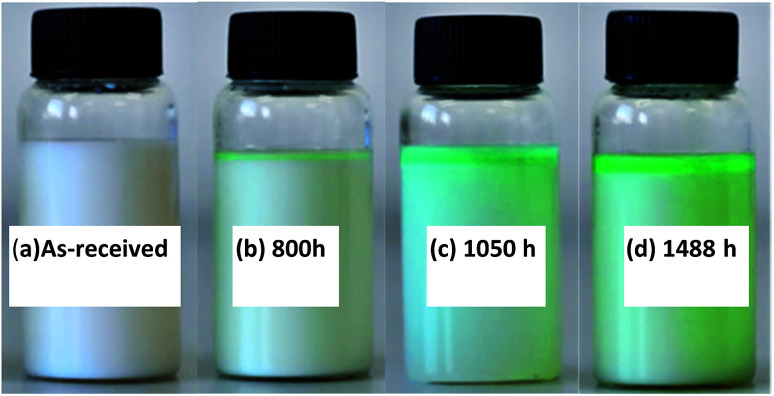
Color of O/W emulsion before (a) and after (b–d) immersing copper for different times.

The corrosive emulsion was collected again for observation. Fig. 9 shows the change in color of the appearance of O/W emulsion for different immersion times with respect to the as-received sample. The original emulsion appeared milky (Fig. 9(a)) and then turned bluish at about 800 h, beginning from the top layer of emulsion (Fig. 9(b)). Copper solute and some amount of copper soap were generated at this period. When the immersion time was over 1050 h (Fig. 9(c)), the presence of blue products increased and the color of emulsion became deep. After 1488 h, the emulsion appeared greenish (Fig. 9(d)). In particular, the emulsion seemed to become unstable with the increase in immersion time and it separated into two layers: the upper layer was the oil phase containing a large amount of copper composites and the bottom layer was the water phase. This phenomenon could be attributed to the fact that surfactants and emulsifiers react with copper and lose the efficiency of forming stable emulsion droplets.

### 3.4 Corrosion mechanism analysis

**Fig. 10:**
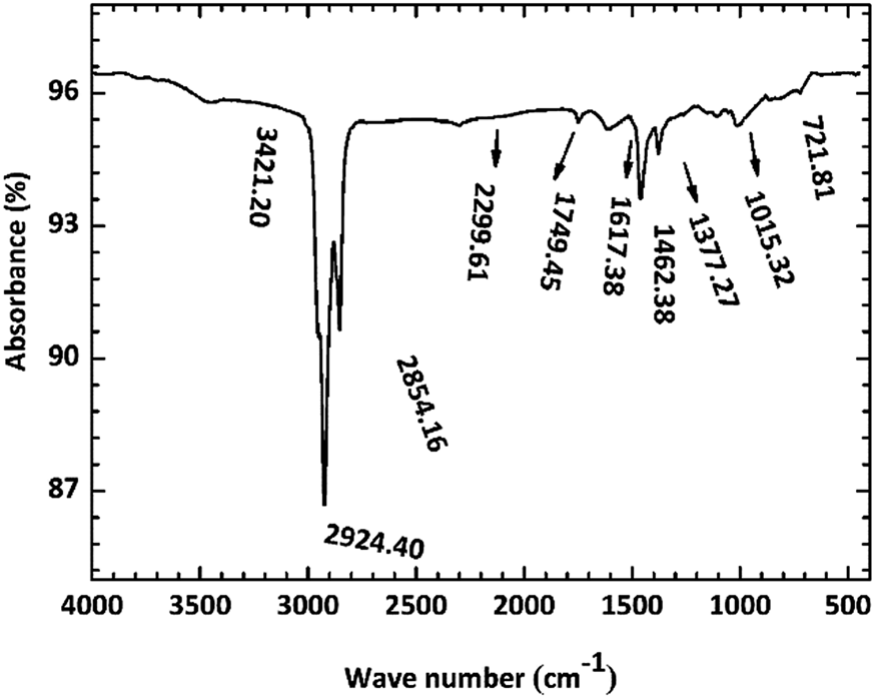
FT-IR spectrum of copper surface after immersion for 1488 h at room temperature.

Fig. 10 shows the FI-IR spectrum of the copper surface after immersion in O/W emulsion for 1488 h. The high intensity peaks at the region of 2924 and 2854 cm^−1^ were assigned to the symmetric stretching vibration bands of –CH_3_ and asymmetric stretching vibration band of –CH_2_, respectively. The peaks at 1377 and 722 cm^−1^ also indicated the presence of long chain saturated hydrocarbons. The low intensity peaks at the 2299 cm^−1^ region represented the stretching vibration of C=C, indicating the presence of unsaturated hydrocarbons. The above results proved that the oil phase aggregates on the copper surface. Furthermore, the peaks at 1462, 1749 and 1015 cm^−1^ seemed to indicate the stretching vibrations of C–C, C=O and C–O, respectively. This region is most likely to be attributed to a saturated ester formed from the saponification reaction or the carboxylate (COO–) anion, which can form metal carboxylate corrosion compounds such as CuCO_3_ moieties,^37^ as mentioned in most articles. The 3421 cm^−1^ band was assigned to the OH– groups, which seemed to indicate the presence of Cu(OH)_2_.

**Fig. 11:**
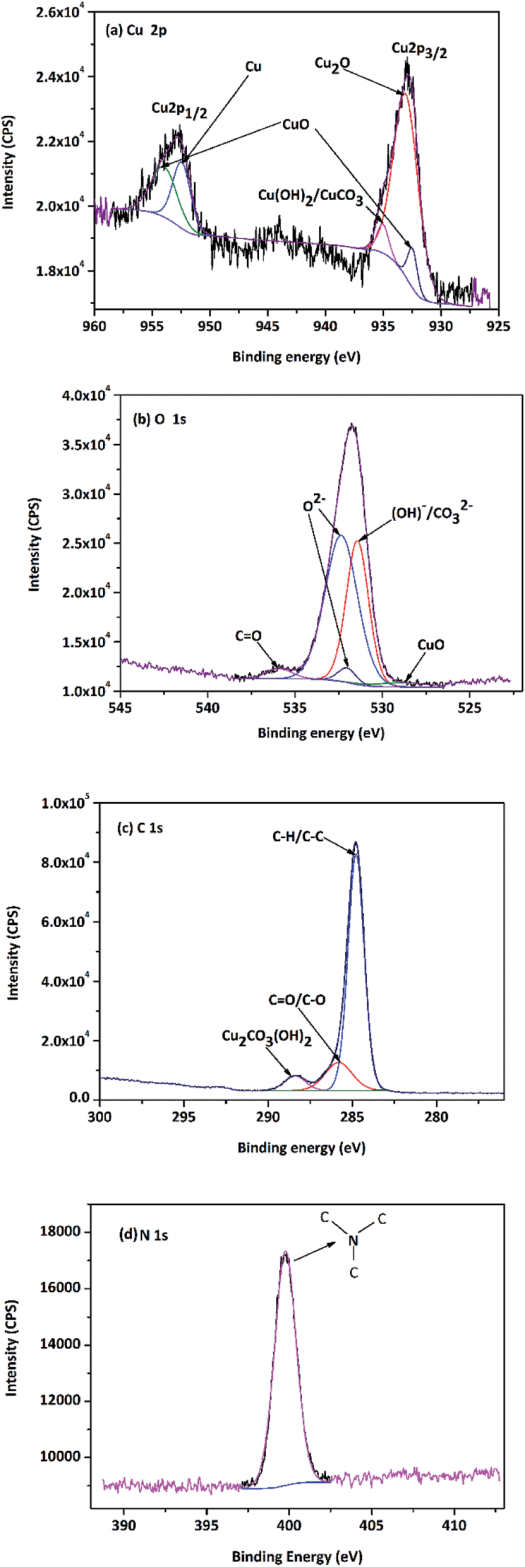
XPS spectra of copper surface after 1488 h immersion test (a) Cu 2p, (b) O 1s, (c) C 1s and (d) N 1s.

To further characterize the compositions of corrosion products on the copper surface, XPS analysis of Cu specimen was performed after the 1488 h immersion experiment. The binding energies of some standard compounds of Cu, O, C and N are listed in Fig. 11, which were obtained from NIST XPS database.

As shown in Fig. 11(a), The Cu 2p XPS peaks at 928–938 and 952–957 eV correspond to Cu 2p_3/2_ and Cu 2p_1/2_, respectively.^37^ The binding energies of 952.6 eV and 933.1 eV represent the presence of metallic Cu state and the Cu_2_O compound. The peaks detected at 953.7 eV and 932.6 eV undoubtedly indicated the presence of CuO. A weak peak at around 935.1 eV showed that other copper compounds Cu(OH)_2_/CuCO_3_ could exist on the specimen surface. Moreover, it was found that the peaks of hydroxyl (OH–) and O^2−^ were located at 531.9 eV and 530.5 eV in O 1s spectrum (Fig. 11(b)); therefore, the specimen surface likely had CuCO_3_/Cu(OH)_2_ as reported by Colin *et al.*^38,39^ Furthermore, a peak at 535.1 eV is indicative of the C=O state. From the C 1s spectrum in Fig. 11(c), the presence of organic carbon indicated by the peak at 284.6 eV and the presence of pseudomonas indicated by the peak at 289.0 eV are also realized. In particular, the peak at 285.6 eV represents the existence of carbonyl group, which could be attributed to the saponification reaction in emulsion. The spectrum of N 1s (Fig. 11(d)) shows only a single binding energy peak at 399.6 eV, corresponding to the saturated N–C in triethanolamine, which indicates that nitrogen is only adsorbed on the copper surface and does not undergo any chemical reaction with the other components in emulsion.

It could be inferred that the surfactants could decompose and form free carboxylate or ester groups. These functional groups could react with copper and generate bulk copper carboxylate together with O_2_, CO_2_ and H_2_O molecules and decomposed ions and finally form Cu(OH)_2_, CuCOO^−^ and even pseudomonas (Cu_2_(OH)_2_CO_3_).

Summarizing the results of corrosion rates, the appearances of copper surfaces and emulsion, and the analysis of surface corrosion products, it is concluded that copper preferentially reacts with O atoms of the surfactants and emulsifiers or the oxygen dissolved in emulsion and form the red product Cu_2_O at the initial stage of corrosion and then convert to CuO with the help of OH^−^ in water and the effect of free hydroxyl groups. These implications are in good agreement with those reported in other studies^40–43^ that investigated the effects of H_2_O, O_2_, atmosphere, metal contact, *etc.* The color of copper surface became dense and eventually turned black. With the increase in immersion time, surfactants and emulsifiers gradually adsorb on copper surfaces and some react to form copper composites such as Cu(OH)_2_, CuCO_3_, and Cu_2_(OH)_2_CuCO_3_. The entire reaction process could be inferred from these equations:

3 2Cu + 1/2O_2_ → Cu_2_O

4 2Cu + O_2_ → 2CuO

5 Cu + *n*H_2_O → Cu^0^_*m*_(OH^−^)_*n*_ + *n*H^+^

6 Cu^0^_*m*_(OH^−^)_*n*_ + (*n* + *m*)H^+^ + *m*/2O_2_ → *m*Cu^2+^ + (*n* + *m*)H_2_O

7
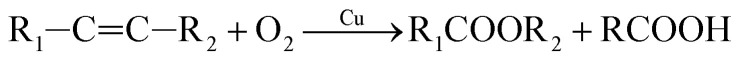

8 4R′COOH + 2CuO → 2Cu(OOCR)_2_ + 2H_2_O

9 Cu^2+^ + 2RCOO^−^ → CuCO_3_ + R–R + CO

10 2Cu + O_2_ + H_2_O + CO_2_ → Cu_2_(OH)_2_CO_3_

**Fig. 12:**
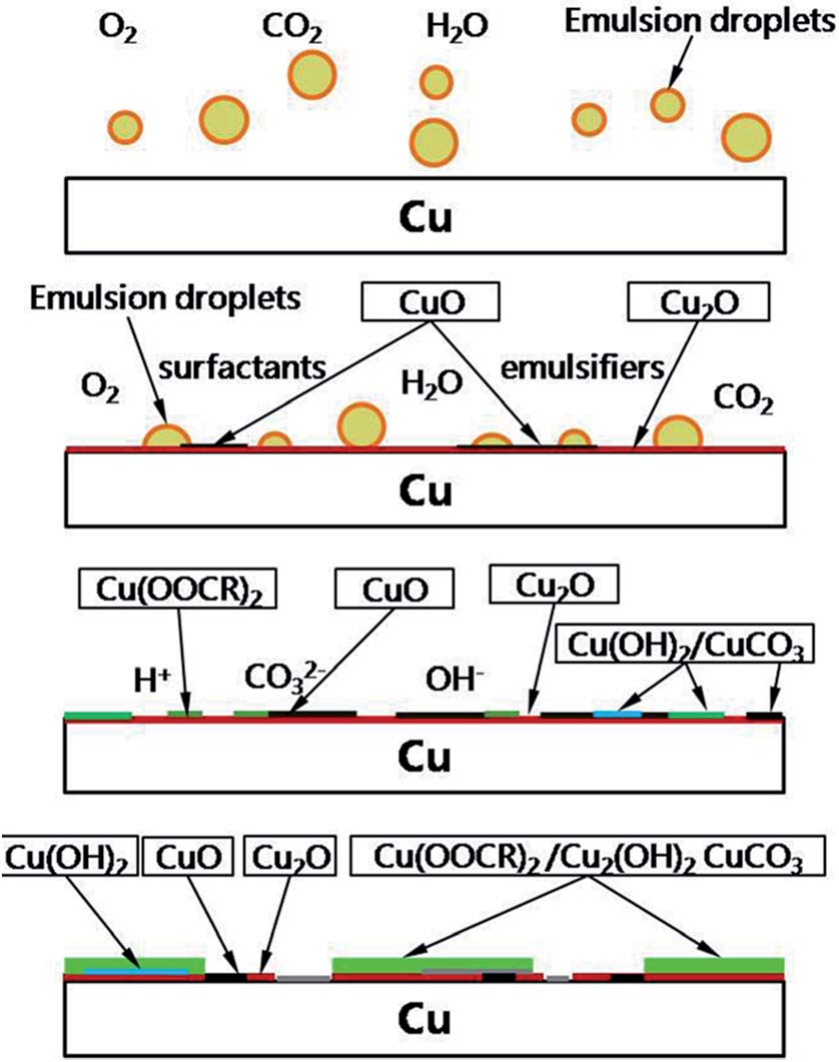
The schematic sketching for the corrosion mechanism of copper in an O/W emulsion.

The mass consumption of surfactants ultimately results in the hierarchy of the O/W emulsion. These insoluble compounds finally suspend into the light oil phase and change the color of emulsion to green or blue. The schematic diagram for the corrosion mechanism of copper in an O/W emulsion is shown in Fig. 12.

## 4. Conclusions

In this study, the corrosion mechanism of copper in the O/W emulsion has been studied through static immersion experiments. Surface analysis and elemental analysis were performed to provide the evidences of the corrosion products and the key findings were the following:

(1) Copper possesses an incubation period on its corrosion behaviour and the corrosion rate of copper in an emulsion exhibited a nearly linear increasing trend at room temperature and the corrosion kinetics relationship of copper could be deduced as *D*_1_ = 2.66 × 10^−3^*t*^11.68^.

(2) The corrosion products seemed to be Cu_2_O, CuO, Cu(OH)_2_, CuCO_3_ and Cu_2_(OH)_2_CuCO_3_, which were formed with the increase in the immersion time. The color change in emulsions was attributed to the copper soaps of these compounds that were distributed in the oil phase. The size of emulsion droplets was found to be 22 μm in average. These liquids adsorb on copper surfaces, causing corrosion pitting and result in the appearance of “emulsion spot” defects.

(3) Copper is more likely to react with O atoms in emulsions to form Cu^+^ and Cu^2+^. Then, they adsorb components that contain hydroxide and carboxylate anions to generate copper compounds. The surfactants were consumed and the efficiency of emulsification characteristics was lost. Finally, O/W emulsion separated into two layers, which might hint the significance of introducing an inhibitor to protect the copper surfaces.

## Conflicts of interest

There are no conflicts to declare.

## Supplementary Material
